# Tissue response and clinical outcomes after cardiovascular use of porcine small intestinal small intestinal submucosal extracellular matrix: a systematic review

**DOI:** 10.3389/fcvm.2025.1532157

**Published:** 2025-06-25

**Authors:** Villads Juul Bruun, Line Lyngbak Jensen, J. Michael Hasenkam, Johannes H. Jedrzejczyk

**Affiliations:** ^1^ Department of Cardiothoracic and Vascular Surgery, Aarhus University Hospital, Aarhus, Denmark; ^2^ Department of Clinical Medicine, Aarhus University, Aarhus, Denmark

**Keywords:** porcine small intestinal submucosal extracellular matrix, small intestinal submucosal extracellular matrix, CorMatrix, cardiovascular surgery, tissue engineering, bio scaffold

## Abstract

**Background:**

Porcine small intestinal submucosal extracellular matrix (PSIS-ECM) is a biomaterial that has gained increasing popularity in cardiovascular surgery over the past three decades. This popularity is due to PSIS-ECM demonstrating properties of an ideal biological scaffold; it is easy to use, lacks immunogenicity, is absorbable, possesses the potential to promote native tissue growth, and exhibits remodelling properties. We systematically reviewed the literature on the preclinical and clinical use of this approach in cardiovascular surgery over the past decade.

**Methods:**

Utilizing a box-search methodology, an extensive survey of the literature on PSIS-ECM's application in cardiovascular surgery from 2013 until September 2023 was conducted within the PubMed and Embase databases. Initially, 245 publications were identified. Following title, abstract, and full-text screening, 66 articles were included in the survey.

**Results:**

Among nine preclinical studies conducting histological assessments of explants, eight did not report signs of inflammation. Tissue remodelling was documented in six preclinical studies. Histological examination of explants was incorporated into thirteen clinical cohort studies, all of which demonstrated varying intensities of inflammation and no or minimal signs of regeneration and remodeling. The reintervention rates among clinical cohort studies range from 4.5% to 87.5%. Eleven studies reported a reintervention rate exceeding 15%, while six reported a reintervention rate below 15%.

**Conclusion:**

Preclinical studies corroborate the notion that PSIS-ECM exhibits properties of an ideal biological scaffold. However, these findings lack reproducibility in clinical settings. Combined with reports from clinical studies showing reintervention rates exceeding 15%, this has raised concerns about whether clinical application of PSIS-ECM should be confined to selected cases.

## Introduction

Cardiovascular disease (CVD) is the predominant cause of global mortality (1), posing a significant challenge to global health. CVD is a heterogeneous group of diseases, including acquired and congenital pathologies. Irrespective of etiology, surgical interventions are often the primary treatment, and the procedures frequently necessitate the implantation of artificial tissue to replace or reconstruct deficient, damaged, or defective tissue. Therefore, throughout the years, several materials, encompassing autologous, xenogeneic, and synthetic materials, have been proposed to fulfill the requirements for various surgical applications. Nonetheless, these materials have both advantages and disadvantages. Biological materials, such as autologous and xenogeneic pericardium, are prone to retraction, fibrosis, thickening, and calcification (2). Synthetic materials inherently lack biocompatibility, exhibit rigidity, and promote reactive inflammation (3). Neither of these materials facilitates tissue growth, which is particularly desirable in pediatric patients. Thus, developing a material that does not exhibit these limitations has long been a sought-after goal.

Porcine small intestinal submucosal extracellular matrix (PSIS-ECM), a decellularized small intestinal submucosa, is a biological scaffold that has become increasingly popular in cardiovascular surgery over the last two decades. The popularity is based on early preclinical studies presenting results that indicate PSIS-ECM exhibits characteristics of an ideal material for cardiovascular tissue engineering. It is easy to handle, resistant to fibrosis and calcification, lacks immunogenicity, and possesses the potential to promote infiltration of host cells and tissue growth.

Since the first clinical application of PSIS-ECM in 2010, it has been utilized for various purposes in cardiovascular surgery. Nezhad et al. have published a comprehensive literature review on the utilization of PSIS-ECM up to 2015 (3). However, as PSIS-ECM had only been in clinical use for five years at the time of their study, the number of included clinical cohort studies in their review is limited. Consequently, we conducted a similar study focusing on more recently published research. Here, we present the results of preclinical and clinical studies on the application of PSIS-ECM over the last decade in both adult and pediatric patients.

## Methods

**Figure 1:**
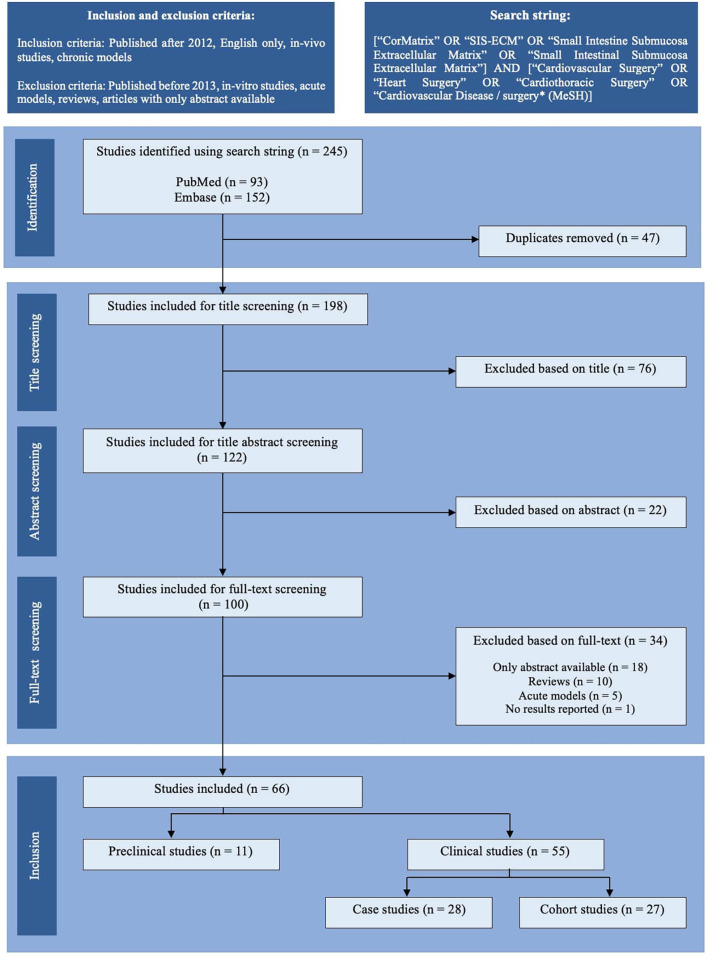
PRISMA-style flow chart of the study selection process. The literature search identified 245 records through PubMed and Embase. After removing 47 duplicates, 198 studies were screened by title, of which 76 were excluded. Of the 122 abstracts screened, 22 were excluded. Following a full-text screening of 100 articles, 34 were excluded due to the unavailability of full text (*n* = 18), reviews (*n* = 10), the use of acute models (*n* = 5), or the lack of reported results (*n* = 1). Ultimately, 66 studies were included: 11 preclinical (*in vivo*) studies and 55 clinical studies, further classified as case studies (*n* = 28) or cohort studies (*n* = 27).

Utilizing a box-search methodology, an extensive literature survey was conducted from 2013 to September 2023, encompassing the PubMed and Embase databases. The search string employed was as follows: [“CorMatrix” OR “SIS-ECM” OR “Small Intestine Submucosa Extracellular Matrix” OR “Small Intestinal Submucosa Extracellular Matrix”] AND [“Cardiovascular Surgery” OR “Heart Surgery” OR “Cardiothoracic Surgery” OR “Cardiovascular Disease/surgery* (MeSH)]”. Figure 1 illustrates a schematic representation of the literature search process, following the PRISMA 2020 flow chart. Two independent authors screened the articles (VJB and LLJ). Only articles chosen by both authors at each screening step proceeded to the next stage. Just the abstracts of articles with either relevant titles or keywords were assessed. The complete content of potentially relevant articles was examined for potential inclusion in the study, resulting in the inclusion and tabulation of 66 articles (Tables 1–3). Any discrepancies in the inclusion of articles were identified and resolved via discussion. The scientific methodological quality of the included articles was evaluated. However, no quantitative rating tool was employed because “quality assessment of observational studies is not typically conducted in systematic reviews” (4).

**Table 1:** Preclinical studies on the utilization porcine small intestinal submucosal extracellular matrix.

Author	Year	Indication	Animal model	Number	Follow-up	Layers in patch	Outcome measure	Result(s)
Toeg et al. (5)	2013	Myocardial infarct	Mice	*n* = 38 (*n* = 8, PBS, *n* = 10, CACs, *n* = 10, PSIS-ECM, *n* = 10, PSIS-ECM + CACs)	28 days	N/A	Echocardiography Histological examination	*Clinical:* The left ventricular wall thickness was significantly greater in PSIS-ECM and PSIS-ECM + CACs groups compared to PBS and CACs groups. Infarct size was significantly reduced in PSIS-ECM and PSIS-ECM + CACs groups. *Histological:* Arteriolar density in periinfarct regions was enhanced in PSIS-ECM and PSIS-ECM + CACs groups. More GATA4- and b-catenin–positive cardiac cells were found in the myocardium of PSIS-ECM and PSIS-ECM + CACs groups.
Fallon et al. (8)	2014	Tricuspid valve replacement	Sheep	*n* = 4	3 months (1 sheep) 5 months (1 sheep) 8 months (1 sheep) 12 months (1 sheep)	2-ply	Echocardiography Histological examination of explants	*Clinical:* Echocardiographic assessment demonstrated that bioprosthetic tricuspid valve function, leaflet mobility, and leaflet thickness were essentially normal in all animals. *Histological:* Massive host-cell infiltration and structural reorganization of the PSIS-ECM bio scaffold. Elastin generation at the annulus by 3 months. Increased collagen organization and glycosaminoglycan presence in the leaflets by 5 months. No evidence of foreign body response.
Mewhort et al. (7)	2014	Myocardial infarct	Rats	*n* = 56 (*n* = 13 normal, *n* = 18 non-enhanced PSIS-ECM, *n* = 10 bFGF-enhanced PSIS-ECM, *n* = 15 sham-procedure)	16 weeks	N/A	Echocardiography and histological examination	*Clinical:* Ejection fraction improved in the bFGF-enhanced PSIS-ECM-group compared to sham (55.3% ± 8.0% vs. 35.1% ± 7.6%; *P* < .001). *Histological:* Histology and immunohistochemistry demonstrated comprehensive integration of PSIS-ECM biomaterial patch without evidence of immune reaction in non-enhanced PSIS-ECM- and bFGF-enhanced PSIS-ECM-group.
Tanaka et al. (13)	2015	Myocardial repair	Pigs	*n* = 15 (Group B, *n* = 5, PSIS-ECM treated with FGF-2; Group U, *n* = 5, untreated PSIS-ECM; Group D, *n* = 5, Dacron)	60 days	N/A	Cardiac MRI Electrophysiologic-Anatomic mapping Histological examination of explants	*Clinical:* CMR–derived parameters were better in group B compared to groups U and D. Electrophysiologic-anatomic mapping in group B indicated positive electrical conductivity in the patch area. *Histological:* Histology in group B showed a homogenous distribution of host cells, including tropomyosin and a-sarcomeric actinin–positive maturing cardiomyocytes. Group B demonstrated the greatest degree of vasculogenesis as determined by capillary density analysis.
Zafar et al. (9)	2015	Tricuspid valve replacement	Lambs	*n* = 12 (*n* = 8, PSIS-ECM, *n* = 2, conventional bioprosthetic valve, *n* = 2 native valves)	3 or 8 months	2-ply	Echocardiography Histological examination of explants	*Clinical:* The PSIS-ECM valves showed a gradual increase in annular diameter similar to native valves. PSIS-ECM valve function was normal in 7 of 8. However, 1 had severe regurgitation due to flail leaflet. *Histological:* Histopathology demonstrated migration of resident mesenchymal cells into the scaffold and trilaminar PSIS-ECM organization similar to a native valve, without inflammation or calcification at 8 months.
Miller et al. (14)	2016	Pulmonary valve replacement	Pigs	*n* = 6	Up to 6 months	2-ply	Echocardiography Histological examination	One pig was euthanized after 3 months for midterm analysis, and another animal died due to endocarditis at 3 months. *Clinical:* The valves increased in size, however, all valves developed at least moderate regurgitation. *Histological:* Histology showed advanced remodelling with myofibroblast infiltration, neovascularization, and endothelialization. There were, nevertheless, some unanticipated findings including thinning of the infundibulum and the infiltration of bacterial colonies in two animals.
Scully et al. (10)	2016	Myocardial repair	Sheep	*n* = 4	5 months or 8 months	N/A	Echocardiography Histological examination of explants	*Clinical:* Epicardial echocardiography showed that the patch contracted with the native myocardium, with thickening of the patch region seen during systole. *Histological:* The patches had undergone extracellular matrix remodelling and neovascularization without calcification. The PSIS-ECM was populated with muscle cells positive for sarcomeric alpha-actinin, CD45, and troponin I and T.
Mewhort et al. (15)	2016	Epicardial Infarct Repair	Pigs	*n* = 12 (*n* = 8 EIR, *n* = 4 sham treatment)	6 weeks	N/A	Cardiac MRI	*Clinical:* The EIR-group exhibited reduced fibrosis, less myocardial restraint, increased wall thickening, and increased vascularity compared to the sham treatment-group.
Wang et al. (6)	2017	Myocardial infarct	Mice	Not specified, although at least *n* = 1 sham-procedure *n* = 1 Myocardial infarct *n* = 1 Myocardial infarct + PSIS-ECM-patch *n* = 1 Myocardial infarct + PSIS-ECM-patch + CPCs	28 days	N/A	Echocardiography and histological examination	*Clinical:* Transplantation of the PSIS-ECM-patch alone increased the LVEF modestly in MI-mice. In contrast, transplantation of PSIS-ECM-patch + CPCs significantly increased the LVEF (*p* < 0.004). Transplantation of PSIS-ECM to MI heart appeared to have modest effect on reducing the scar area. In contrast, PSIS-ECM with CPC seeding significantly reduced the scar area compared with the MI-group.
Baker et al. (11)	2019	Right ventricular wall defect	Juvenile sheep	*n* = 11	Up to 12 months	4-ply	MRI Three-dimensional-electrophysiologic mapping Histological examination of explants	*Clinical:* MRI revealed changing strain properties of the patch which by 9–12 months resembled native tissue. Three-dimensional mapping demonstrated consistent conduction across the patch with minimal voltage differences. However, the conduction velocity was consistently less than native myocardium. *Histological:* The PSIS-ECM-patches were consistently populated with viable cells, mainly fibroblasts, smooth muscle cells, adipocytes, and endothelial cells. However, it remained histologically immature. Minimal to no calcification was observed.
Van Rijswijk et al. (12)	2020	Pulmonary valve replacement	Sheep and lambs	*n* = 10 sheep *n* = 10 lambs	1 month (3 sheep and 3 lambs) 3 months (3 sheep and 3 lambs) 6 months (4 sheep and 4 lambs)	N/A	Echocardiography Histological examination of explants	*Clinical:* 5 sheep and 2 lambs died due to congestive heart failure within 2 months after surgery (before follow-up ended). 5 sheep and 8 lambs survived follow-up. 5 lambs had significant pulmonary stenosis and 1 sheep showed severe regurgitation. The rest had well-functioning valves. *Histological:* In animals that died before follow-up, the valve leaflets were thickened with signs of inflammation (endocarditis in 4). Among animals surviving until follow-up, signs of chronic inflammation, fibrosis, and limited signs of remodelling were found.

Scaffold source was not consistently reported and is therefore omitted from this table. See discussion for further elaboration.

N/A, not applicable; PBS, phosphate buffered saline; CACs, circulating angiogenic cells; PSIS-ECM, porcine small intestinal submucosal extracellular matrix; bFGF, basic fibroblast growth factor; FGF-2, basic fibroblast growth factor; MRI, magnetic resonance imaging; EIR, epicardial infarct repair; LVEF, left ventricular ejection fraction; MI, myocardial infarction; CPCs, cardiac progenitor cells.

**Table 2:** Clinical case studies on the utilization of porcine small intestinal submucosal extracellular matrix.

Author	Year	Indication	Patient(s)	Follow-up	Layers in patch	Outcome measure	Result(s)
Stelly and Stelly (16)	2013	Pericardial closure	*n* = 1 60-yo man	5 years	N/A	Histological examination after reintervention	*Histological:* Clear evidence of constructive remodelling into viable, non-fibrotic connective tissue with no evidence of any destructive inflammatory response.
Yeen et al. (28)	2013	Anomalous pulmonary vein reconstruction	*n* = 1 18-yo woman	3 months	N/A	CT-angiography	*Clinical:* The patient was reoperated 10 days after surgery due to a clot found near patch. No patch-failure reported.
Eckhauser et al. (21)	2013	Innominate artery repair	*n* = 1 12-yo child	8 days	N/A	MRI	*Clinical:* The repair was intact at discharge after 8 days
Yanagawa et al. (39)	2014	False aneurysm repair, myocardial repair	*n* = 1 75-yo woman	1 year	N/A	Echocardiography	*Clinical:* Restored left ventricular function with normalization of ejection fraction (from 20 to 40% to >60%). The area of the PSIS-ECM-patch appeared to have increased wall thickness and synchronous contractile activity.
Slachman (30)	2014	Aortic root repair	*n* = 1 90-yo woman	34 months	N/A	Autopsy, histological examination	*Histological:* Constructive remodelling and host tissue regeneration at the site of PSIS-ECM-patch. Limited or minor evidence of inflammation and calcification was seen at the margins of the patch.
Deorsola et al. (22)	2014	Aortic coarctation	*n* = 1 3-weeks-old child	1 year	N/A	Echocardiography	*Clinical:* 4–5 months after surgery the patient had restenosis and interventional cardiology procedure with a stent was performed. No further events were seen the next 6 months.
Sündermann et al. (40)	2014	Endocarditis	*n* = 2 75-yo man 47-yo man	34 days and 3 months	N/A	Echocardiography	*Clinical:* At follow-up, both patients presented well-functioning valves.
Wallen et al. (41)	2014	Endocarditis of tricuspid valve	*n* = 1 53-yo man	3 months	N/A	Echocardiography	*Clinical:* Successful repair with mild residual tricuspid regurgitation.
Madden et al. (29)	2014	Carotid Endarterectomy	*n* = 1 71-yo woman	N/A	N/A	Ultrasonography	*Clinical:* Successful repair.
Toyoda et al. (42)	2014	Endocarditis of tricuspid valve	*n* = 1 34-yo woman	N/A	N/A	Echocardiography Histological examination of explant	*Clinical:* Tricuspid valve regurgitation; the entire PSIS-ECM tube prolapsed into the right atrium. *Histological:* Tissue with necrosis, fibrinopurulent debris, gram-positive bacterial colonies, and granulation tissue formation.
Brinster and Patel (35)	2014	Aortic Root Enlargement	*n* = 7	N/A	N/A	Echocardiography	*Clinical:* All patients had successful aortic root enlargement. No failures reported.
Szczeklik et al. (31)	2015	Reconstruction of superior vena cava and right atrium	*n* = 1 47-yo woman	8 weeks	N/A	Echocardiography	*Clinical:* Successful reconstruction with no complications.
Abu Saleh et al. (32)	2015	Replacement of an ascending aorta graft	*n* = 1 64-yo man	14 months	N/A	CT-scan, reoperation, and histological examination of explant	*Clinical:* CT-scan showed formation of a pseudoaneurysm at the PSIS-ECM-graft location. *Histological:* Compared to an unused PSIS-ECM-patch, the PSIS-ECM-explant was almost identical in terms of diameter, cellularity, or organization. Thus, no remodelling had occurred.
Holubec et al. (38)	2015	Post-infarction left ventricular free wall rupture	*n* = 1 50-yo man	3 months	N/A	Echocardiography	*Clinical:* Successful repair with normal left ventricle size and left ventricle ejection fraction at 50%.
Bibevski et al. (17)	2015	Atrioventricular valve replacement in patient with hypoplastic left heart syndrome	*n* = 1 4-month-old infant	25 days	2-ply	Echocardiography Autopsy, examination of valve	*Clinical:* Echocardiography showed excellent function of the valve. *Histological:* Very little cellular infiltration. There was layering of macrophages.
Luk et al. (43)	2015	Endocarditis of PSIS-ECM-patch	*n* = 2 56-yo and 46-yo man	10 months and 18 months	N/A	Histological examination of explant	*Histological:* No evidence of cellular infiltration in the tissue itself was found in either case, though there was mild inflammation seen at anastomosis sites. Leukocytes, macrophages, stem cells, and new blood vessels were seen only at the anastomosis sites.
Fernandez-Doblas et al. (23)	2016	Congenital heart defects, various sites	*n* = 30 Mean age at surgery 6 months (1 month–3.8 years)	Mean 268 days (194.5–305.8 days)	N/A	Serial echocardiography	*Clinical:* Two patients required reoperation during follow-up. One due to stenosis after pulmonary artery plasty. The other due to tricuspid stenosis secondary to thickening of the septum caused by an excessive inflammatory response secondary to foreign body reaction.
Guariento et al. (18)	2016	Atrioventricular valve regurgitation in patient with functionally univentricular heart	*n* = 1 128 days old child	1 year	4-ply	Echocardiography, reoperation, and histological examination of explant	*Clinical:* Severe right atrium dilatation, no AVV regurgitation, but a thickened PSIS-ECM tissue, with AVV stenosis. *Histological:* Fibroblast hyperplasia on the atrial and ventricular side associated with fibrosis, an intense chronic inflammatory infiltrate, no signs of acute damage, and no calcifications.
Khan et al. (24)	2016	Left coronary artery occlusion and dilated left ventricle	*n* = 1 7 months old child	18 months	N/A	CT-angiogram	*Clinical:* At follow-up, the angiograms revealed an unobstructed LCA system and normal LV size and function.
Nezhad et al. (19)	2017	Aortic valve stenosis repair	*n* = 1 12-yo child	4 years	4-ply	Echocardiography, reoperation, and histological examination of explant	*Clinical:* At 52,5 months echocardiography showed patch prolapse with severe regurgitation, mild stenosis, and left ventricle dilatation. *Histological:* Native cusp had chronic inflammation with lymphocytes and macrophages. PSIS-ECM-cusp was thickened, fibrotic and calcified. Moderate chronic inflammation as well as subacute inflammation was found.
Ferng et al. (33)	2017	Reconstruction of aortic graft and ventricular apex defect	*n* = 1 62-yo man	3 months	N/A	Histological examination of explant	*Histological:* After 3 months *in vivo*, histologic examination revealed cardiac tissue growing on the endoluminal apical PSIS-ECM material. Furthermore, cardiac striations similar to native cardiac tissue were observed. Biopsy specimens of the aorta PSIS-ECM showed elastin and collagen growth patterns comparable to those found in normal aortic histologic patterns.
Ali and Dunning (34)	2018	Superior vena cava obstruction	*n* = 5	Median 16 months	N/A	Reintervention rate	*Clinical:* No episodes of stenosis or thrombosis, and no need for reintervention in any patient.
Szalanski et al. (20)	2019	Significant mitral and tricuspid regurgitation	*n* = 1 43-yo man	52 days	N/A	Echocardiography and reoperation	*Clinical:* Echocardiography 11 days after surgery showed paravalvular leak at the mitral prothesis. Reoperation was performed and the leak was repaired. The following post operative period was without issues, and 15 days later (52 days after first surgery) the patient was discharged.
Rodrigues et al. (25)	2020	Tetralogy of Fallot and hypoplastic intrapulmonary pulmonary arteries	*n* = 1 5-yo child	1 month	N/A	Echocardiography, CT-angiogram, reoperation, and histological examination of the tissue.	*Clinical:* Echocardiography at 1 month presented stenosis of the right and left PA. CT-angiogram showed giant pseudoaneurysm causing the stenosis. Reoperation revealed that the patch had almost disintegrated. *Histological:* Histology of the pseudoaneurysm-wall showed fragments of patch-material engulfed in fibrosis, eosinophils, and giant multinuclear cells.
Bibevski et al. (26)	2020	Transposition of the Great Arteries	*n* = 1 11-month-old child	11 months	2-ply	Histological examination of explant	*Histological:* Collagen deposition with re-endothelization of the surface. Mild inflammation was found.
Mously et al. (37)	2021	Ventricular septal defect secondary to myocardial infarct	*n* = 1 58-yo woman	7 months	N/A	Echocardiography and reoperation	*Clinical:* Near-complete reabsorption and early failure of patch.
Burke et al. (27)	2022	Tetralogy of Fallot (PSIS-ECM used for RVOT-patch)	*n* = 1 29-yo woman	5 years	N/A	Reoperation	*Clinical:* 5 years after operation using PSIS-ECM as patch for RVOT-defect, the patient presented with decreased right ventricular function. At reoperation dehiscence of the RVOT patch from the anterior valve sewing ring was found. There was no evidence of infection.
Bhatt et al. (36)	2023	Myocardial infarct	*n* = 1 29-yo man	14 months	N/A	Late gadolinium enhancement cardiac magnetic resonance	*Clinical:* Left ventricular function improved. Ejection fraction changed from 10% pre-operation to 51% after 14 months.

Scaffold source was not consistently reported and is therefore omitted from this table. See discussion for further elaboration.

N/A, not applicable; Yo, years old; CT, computed tomography; PSIS-ECM, porcine small intestinal submucosal extracellular matrix; MRI, magnetic resonance imaging; AVV, atrioventricular valve; LCA, left coronary artery; LV, left ventricle; PA, pulmonary artery; RVOT, right ventricular outflow tract.

**Table 3:** Clinical cohort studies on the utilization of porcine small intestinal submucosal extracellular matrix.

Author	Year	Indication	Patients	Age at surgery	Follow-up	Layers in patch	Outcome measure	Result(s)
Yanagawa et al. (44)	2013	Cardiac complications after myocardial infarction	*n* = 11 (*n* = 7, ventricular aneurysm, *n* = 3 ventricular septal defect, *n* = 1 both)	Mean: 67 ± 11 years (52–83 years)	Mean 204 days (4–642 days)	N/A	Echocardiography	*Clinical:* Two reoperations and one surgical mortality, none of which were related to PSIS-ECM failure.
Witt et al. (45)	2013	Congenital heart defects, various sites	*n* = 48 patches (37 patients) *n* = 13 septal defects *n* = 26 vascular augmentation *n* = 7 outflow tract augmentation *n* = 3 valve reconstruction	Mean: 18.2 ± 47.7 months	Mean 411 days (6–757 days)	N/A	Echocardiography Histological examination of explants	*Clinical:* Four deaths, none related to PSIS-ECM-patch. 2/37 (5.5%) required reintervention related to PSIS-ECM-patch. One due to RVOT obstruction, and one due to stenosis of the left pulmonary artery. A third patient required reintervention unrelated to PSIS-ECM-patch. *Histological:* Signs of chronic inflammation, eosinophilia, and fibrosis.
Zaidi et al. (46)	2014	Valvuloplasty of the aortic and/or mitral valve in patients with congenital heart defects	*n* = 57 (*n* = 25 mitral valve, *n* = 27 aortic valve, *n* = 5 both valves) Available for histology: *n* = 9, PSIS-ECM, *n* = 9, autologous pericardium	19 days–16.7 years	Median days *in situ* for PSIS-ECM explants (mitral valve): 64 (5–261) Median days *in situ* for autologous pericardium (mitral valve): 1010 (14–1,506) Median days *in situ* for PSIS-ECM explants (aortic valve): 63 (49–198) Median days *in situ* for autologous pericardium (aortic valve): 1,747 (6–6,047)	N/A	Histological examination of explants	*Histological:* Chronic inflammation with giant cells surrounded by lymphocytes, macrophages, plasma cells, and eosinophils were seen in 8/9 (88.9%) PSIS-ECM-explants. Mild cellular infiltration of PSIS-ECM was noted in all cases; however, in no case, did it appear that PSIS-ECM was being remodeled into tissue resembling a 3-layered native valve. In contrast, a near absence of any inflammatory reaction was seen and no eosinophilia associated with autologous pericardium was present. Furthermore, more tissue infiltration, remodelling, vascularization, and neointima formation was observed with autologous pericardium.
Gerdisch et al. (48)	2014	Tricuspid valve repair after infectious endocarditis	*n* = 18	Mean: 34.8 ± 8.8 years	1–18 months	N/A	Echocardiography	*Clinical:* No deaths observed. 3/18 (16.6%) required reoperation. Two due to tear of papillary attachment. One patient was reoperated due to worsening of regurgitation.
Gerdisch et al. (47)	2014	Mitral valve repair	*n* = 19	29–73 years	Median 10.9 months (4 days–48 months)	N/A	Echocardiography	*Clinical:* One early death and two late deaths, none related to the mitral valve repair. Two patients with a history of cancer and chemotherapy required reintervention due to failure of the initial mitral valve repair. Echocardiography during follow-up showed evidence of only mild leaflet thickening, zero to mild regurgitation, and no development of stenosis. No echocardiographic evidence of calcification of the PSIS-ECM patch was identified in any patient.
Rosario-Quinones et al. (49)	2015	Congenital heart defects, various sites	*n* = 25 (*n* = 6 explants available)	5 days–57 years	N/A PSIS-ECM *in situ*: 9 weeks–13 months	N/A	Echocardiography, reoperation, and histological examination of explants	*Clinical:* 6/25 (24%) patients, all 6 pediatric, required reoperation. All had hemodynamically lesions at the site of PSIS-ECM implantation. *Histological:* Intense inflammatory reaction (eosinophils, histiocytes, and plasma cells, with accompanying granulation tissue and fibrosis) was seen in all explants.
Hibino et al. (50)	2015	Central pulmonary artery reconstruction in comprehensive stage II surgery for hypoplastic left-sided heart syndrome. Aortic arch reconstruction	*n* = 13 (*n* = 10, central PA reconstruction and *n* = 3, AA reconstruction)	N/A, pediatric	Central PA reconstruction follow-up: Mean 9.7 months. AA reconstruction follow-up: 2.6–18 months (mean 10.6 months)	N/A	Tubular diameter of PSIS-ECM Interventional procedure	*Clinical:* Central PA reconstruction: Two patients died suddenly at home due to unknown causes after surgery. 8/10 (80%) showed progressive significant stenosis, requiring balloon angioplasty and stent placement. AA reconstruction: No stenosis, no dilatation, and no aneurysm formation in any of the three patients.
Padalino et al. (68)	2015	Congenital heart defects, various sites	*n* = 132 patches (103 patients) *n* = 38 valve repair *n* = 16 septal defects *n* = 71 arterial plasty *n* = 7 other use	Mean: 19.7 months (1 day–62 years)	Median 23.3 months (0.3–55.2 months)	N/A	Reoperation, interventional cardiology procedure, functional failure, and histological examination of explants	*Clinical:* Four late deaths, none related to PSIS-ECM-patch. 19/103 (18.4%) patients underwent reoperation, 6/19 (31.5%) related to PSIS-ECM-patch. 11/103 (10.6%) patients underwent interventional cardiology procedure, 8/11 (72.7%) related to PSIS-ECM-patch. *Histological:* Inflammatory cells, lymphocytes, and some giant cells were found, but no signs of regeneration were seen. No signs of calcification.
Du Bose et al. (70)	2015	Arteriovenous fistula aneurysm	*n* = 15 patients (18 AVFAs) *n* = 2 radiocephalic *n* = 1 brachiobasilic *n* = 15 brachiocephalic	Mean: 49 years (27–80 years)	Mean 6.9 months	N/A	Ultrasound examination and complications	Successful repair in all patients. Two patients experienced events with thrombosis, not related to the patches. 5/15 (33.3%) patients underwent follow-up ultrasound examination at a mean of 6 weeks, and all cases demonstrated patency of the patch without stenosis.
Woo et al. (51)	2016	Congenital heart defects, various sites	*n* = 532 (*n* = 12 explants from 11 patients)	Mean = 5.8 years (6 months–18.2 years)	2.5 years PSIS-ECM *in situ*: 77–1294 days (mean 518.6 days)	N/A	Histological examination of explants	*Clinical:* Six cases (Five patients) showed clinical evidence of graft failure prior to re-surgery. *Histological:* PSIS-ECM was not resorbed, and no cases showed signs of remodelling by integration of native tissue. Chronic inflammation: 11/12 (91.7%) explants. Acute inflammation: 3/12 (25%) explants. Fibrosis in surrounding native tissue: 6/12 (50%) explants. Calcification: 3/12 (25%) explants. Giant cell reaction: 8/12 (66.7%) explants. Neovascularization: 5/12 (41.7%) explants. Degeneration of PSIS-ECM: 9/12 (75%) explants. Necrosis of surrounding tissue: 5/12 (41.7%) explants.
Nelson et al. (52)	2016	Hypoplastic Left Heart Syndrome	*n* = 12 (*n* = 10 explants)	4–8 months	N/A PSIS-ECM *in situ*: 18–26 months (mean 21 months)	4-ply	Histological examination of explants	*Histological:* Fibrosis, acellular material, chronic inflammation, and foreign body giant cell reaction were seen in all explants. Calcification was seen in two explants, elastic fibers were seen in two explants, and eosinophils were seen in two explants. There was no evidence of native tissue ingrowth or regeneration.
Padalino et al. (69)	2016	Semilunar valve repair in children	*n* = 22 *n* = 4, Group 1 (Aortic valve repair) *n* = 18, Group 2 (Pulmonary valve repair) *n* = 2 available for histological examination	Median age Group 1: 88.2 months (34.7–143 months) Median age Group 2: 4.33 months (2.3–167.7 months)	Group 1: Median 18.15 months (6.3–34.8) Group 2: 22.53 months (4.3–51.3)	N/A	Echocardiography, reoperation and/or interventional cardiology procedure, and histological examination of explants	*Clinical:* In Group 1, 2/4 patients (50%) required reoperation, while in Group 2, 3/18 patients (11%) required an interventional cardiology procedure. Furthermore, 11/18 patients (61%) in Group 2 showed increased PV-regurgitation at follow-up. *Histological:* Inflammation with monocytic cells (e.g., macrophages), giant cells, and fibrosis was found. Small signs of calcification were present as well.
Padalino et al. (69)	2016	Pulmonary valve reconstruction	*n* = 34 (*n* = 13 PSIS-ECM, *n* = 21 bovine pericardium)	Median age PSIS-ECM-group: 114 days (65–409 days) Median age bovine pericardium-group: 102 days (69–454 days)	23 months	N/A	Echocardiography	*Clinical:* Early postoperative PV regurgitation was significantly lower in PSIS-ECM-patients at discharge (*p* = 0.0027). However, echocardiographic evaluation detected increased PV regurgitation in PSIS-ECM-group, which was not statistically different from bovine pericardium-patients at follow-up.
Hofmann et al. (53)	2017	Aortic valve stenosis or regurgitation	*n* = 6 (*n* = 4 stenosis, *n* = 2 regurgitation)	2 months–14 years	3 months–3 years	N/A	Echocardiography, reoperation, and histological examination of explants	*Clinical:* 4/6 (66.7%) had reoperation due to sudden valve dysfunction in the form of regurgitation and left ventricular dilatation. Another patient also required reoperation and had this planned, however not yet done at the time of publication. *Histological:* Signs of chronic inflammation involving lymphocytes, plasma cells, foreign body giant cells. Calcification was also seen. No new vascularization was observed, nor was remodelling seen.
Dobrilovic et al. (54)	2017	Femoral artery repair due to claudication	*n* = 7	Median: 67 years	Median 56 months	N/A	Ultrasound imaging, angiography, and reoperation	3/7 (43%) patients experienced significant early complications. Two patients experienced patch rupture and 1 developed a pseudoaneurysm.
Ashfaq et al. (55)	2017	Complete atrioventricular septal defects	*n* = 15	Mean: 205 ± 138 days (82–602 days)	Mean 1,364 days	N/A	Mortality, reoperation, and echocardiography	No mortalities observed. Changes from baseline echocardiography to follow-up: 8/15 (53%) showed no change, 4/15 (27%) showed improvement, and 3/15 (20%) showed a decline in left AV valve function. Of the three patients showing a decrease in left AV valve function, 2/15 (13%) declined from none to mild regurgitation (13%) and 1/15 (7%) from mild to severe regurgitation which required reoperation.
Van r et al. (56)	2017	Anterior leaflet augmentation of the mitral valve	*n* = 44 (*n* = 25 PSIS-ECM, *n* = 19 autologous pericardium) *n* = 4 SIS-ECM-explants available for histology	Mean: 62.6 ± 12.2 years	Up to 12 months	N/A	Echocardiography, reoperation, and histological examination of explants	*Clinical:* 8/25 (32%) of the patients in the PSIS-ECM-group had recurrence of severe mitral regurgitation. 7/8 (87.5%) of these patients underwent reoperation. None of the patients operated with autologous pericardium developed severe mitral regurgitation or required reoperation. *Histological:* Intense acute and chronic inflammation was observed at the periphery of the graft. The inflammatory area was abundantly populated with macrophages, multinucleated giant cells, numerous eosinophils, and granulation tissue. No significant calcification was identified. No evidence of host infiltration was found.
Al Haddad et al. (57)	2018	Complete atrioventricular canal	*n* = 73	Mean: 21.8 ± 22.7 weeks (2.5–184.4 weeks)	Mean 1.6 years	N/A	Echocardiography and reoperation	*Clinical:* One patient died due to respiratory failure, not related to the cardiac repair. 7/73 (9%) patients required reoperation due to cardiac-related indications. Five for left atrioventricular valve repair, one for left atrioventricular replacement and two for isolated residual ventricular septal defect.
Cox et al. (58)	2019	Congenital heart defects, various sites	*n* = 10	1 week–6 years	No follow-up	N/A	Autopsy, histological examination	*Histological:* Development of neo-intima: Endothelial and smooth muscle cells were seen spreading onto the surface of the PSIS-ECM-graft. Mild inflammation with mononuclear cells was seen at the interface between PSIS-ECM and native tissue. Authors state that inflammation is variable and dependent on anatomical location of PSIS-ECM.
Heinisch et al. (59)	2019	Congenital heart defects, various sites	*n* = 4	9 days–41 years	No follow-up	N/A	Histological examination of explants	*Histological:* No evidence of resorption or relevant repopulation with resident cells nor formation of functional tissue structures. In contrast, a mixed chronic inflammatory infiltration, early signs of calcification, and scarring as well as focal pseudocartilaginous transformation were found.
Kiper et al. (60)	2020	Mitral valve repair or replacement	*n* = 8 (*n* = 3 explants available)	5.6 ± 1.6 months	3–24 months	2-ply	Echocardiography, reoperation, and histological examination of explants	*Clinical:* One patient died due to respiratory insufficiency and pulmonary hypertension 3 months after surgery. 6/8 (75%) required reoperation due to worsening of valve stenosis and/or regurgitation (*n* = 4) or acute dehiscence from the papillary muscles (*n* = 2). *Histological:* Calcification, inflammation, and sparse ingrowth of native connective tissue cells into the valve matrix was found in one explant. Foreign body reaction was observed in the other two explants. These valves had no evidence of native connective tissue cell ingrowth into the valve matrix.
Hu et al. (61)	2021	Aortic valve repair	*n* = 38 (*n* = 8 PSIS-ECM, *n* = 30 autologous pericardium)	PSIS-ECM-group: 13.63 ± 6.5 years Autologous pericardium-group: 10.25 ± 6.56 years	5 years	N/A	Freedom from reoperation	*Clinical:* In the PSIS-ECM-group, 1/8 (12.5%) of the patients were free from reoperation. The remaining 7/8 (87.5%) were reoperated due to severe aortic regurgitation caused by retraction, thickening, and stiffening of the cusps. In the autologous pericardium group 19/30 (62.5%) of the patients were free from reoperation. The remaining 11/30 (37.5%) were reoperated due to moderate to severe aortic regurgitation.
Ebert et al. (62)	2021	Pulmonary artery reconstruction	*n* = 214 sites (180 patients) *n* = 37, PSIS-ECM *n* = 44, pericardial bovine *n* = 92, branch patch homograft *n* = 41, autologous pericardium	Median: 12.1 months (5.6–59.5 months)	Median 3.7 years (0.01–15.68 years)	N/A	Reintervention rate	*Clinical:* 6/37 (16.2%) in PSIS-ECM-group, 8/44 (18.2%) in pericardial bovine-group, 17/92 (18.5%) in branch patch homograft-group, and 3/41 (7.3%) in autologous pericardium-group required reintervention. Based on the chi-square test, there was no statistically significant difference in reintervention rate between the different patch-groups (*P* = 0.41).
Sood et al. (63)	2021	Hypoplastic Left Heart Syndrome	*n* = 11 (*n* = 9 explants available)	N/A, children older than 3 months	Median time *in vivo* 4.9 months (4–10 months)	2-ply	Histological examination of explants	*Histological:* Acellular material, chronic inflammation, fibrosis, and foreign body giant cell reaction were seen in all explants. Focal calcification and eosinophils were less frequent findings. No explants demonstrated evidence of native endothelial tissue.
McCready et al. (64)	2021	Carotid Endarterectomy	*n* = 275 patients (291 carotid endarterectomies)	Mean: 70.1 years (48–91 years)	Median 72 months (49–85 months)	6-ply	Computed tomography angiography and reintervention	*Clinical:* 13/275 (4.5%) patients required reintervention due to severe recurrent stenoses. 1/275 (0.3%) patients developed a pseudoaneurysm.
Rescigno et al. (65)	2021	Anterior Leaflet Augmentation of Tricuspid Valve	*n* = 19	65.5 ± 13.5 years	Mean 2.1 years (±1.9 years)	N/A	Echocardiography	*Clinical:* Two in-hospital deaths were recorded; none related to PSIS-ECM-patch. Three late deaths were recorded, two due to cardiovascular causes. At the last follow-up 10/13 (77%) of the survivors had none or mild tricuspid regurgitation while 3/13 (23%) had moderate or severe tricuspid regurgitation.
Weis et al. (66)	2022	Group A – Septal repair Group B – Arterial vessel reconstruction Group C – Valve reconstruction Group D – Venous vessel plasty or atrial reconstruction	*n* = 57 (Group A: *n* = 28 (49%), Group B: *n* = 21 (37%), Group C: *n* = 4 (7%), and Group D *n* = 4 (7%))	Median: 11 months (2 days - 60 years)	Median 43 months (3–64 months)	N/A	Functional failure, defined as patch-related event: Group A —Hemodynamically relevant residual shunt Group B —Vessel/right ventricular outflow tract (RVOT) stenosis or aneurysm Group C — Valve failure defined trough hemodynamically relevant valve stenosis or regurgitation Group D — Any unexpected event related to the patch material. Patch-related redo surgery or interventional cardiology procedure	*Clinical:* Functional failure occurred in 21 patients (36.8%). 2/28 (7.1%) in Group A, 14/21 (66.7%) in Group B, 3/4 (75%) in Group C and 2/4 (50%) in Group D. 18 patients (31.5%) needed reinterventions. 10 patients (17.5%) needed further surgery, six (10.5%) due to a PSIS-ECM scaffold failure: four in Group B and two in Group C. Eight patients (14%) required an interventional cardiology procedure, six (10.5%) were related to PSIS-ECM: four in Group B and two in Group C.
Weis et al. (66)	2022	Corrective repair of Tetralogy of Fallot or an Arterial Switch Procedure in patients born with transposition of the great arteries	*n* = 54 (*n* = 18 using PSIS-ECM, *n* = 36 using AP)	PSIS-ECM-group: Mean 6 months (0–42 months) AP-group: Mean 6.4 months (0–72 months)	47 months (3–64 months) for PSIS-ECM group 121 months (0–203 months) for AP group	N/A	Reintervention rate	*Clinical:* 10/18 (55.5%) patients in the PSIS-ECM group required reintervention. 7/10 (70%%) was due to PSIS-ECM scaffold failure. 11/36 (30.6%) patients required reintervention in the AP group. 3/11 (27.3%) was due to AP patch failure.
Haney et al. (67)	2022	Congenital heart defects, various sites	*n* = 408 patches (309 patients) *n* = 314 arterioplasties *n* = 22 venoplasties *n* = 63 intracardiac repairs *n* = 9 valve repairs	4-ply-group: 11 months (0–533 months) 2-ply-group: 4 months (0–19 months)	Median 3.9 years (3 days–7.4 years)	*n* = 376, 4-ply *n* = 32, 2-ply	Reintervention rate	10/408 (2.5%) required surgical reintervention. 27/408 (6.6%) required percutaneous intervention. Between four-ply and two-ply SIS-ECM, the rate of surgical [2.1% [*n* = 8] vs. 6.3% [*n* = 2], *P* = 0.18] or percutaneous reinterventions [6.4% [*n* = 24] vs. 9.4% [*n* = 3], *P* = 0.46] was not different.

Scaffold source was not consistently reported and is therefore omitted from this table. See discussion for further elaboration.

N/A, not applicable; PSIS-ECM, porcine small intestinal submucosal extracellular matrix; RVOT, right ventricular outflow tract; PA, pulmonary artery; AA, aortic arch; PV, pulmonary valve; AV, atrioventricular; AP, autologous pericardium.

## Results

Preclinical trials evaluating the outcomes of utilizing PSIS-ECM in cardiovascular surgery have been conducted since the early 2000s (3). Over the past decade, PSIS-ECM has been employed in experimental cardiovascular surgery involving various animal models, including mice (5, 6), rats (7), sheep, lambs (8–12), and pigs (13–15). The application of PSIS-ECM has been diverse, including tricuspid valve replacement (8, 9), pulmonary artery replacement (12, 14), and myocardial repair post-infarction (5–7, 10, 11, 13, 15). The results of these studies are presented in Table 1. A recent strategy involves augmenting PSIS-ECM by incorporating growth factors or cardiac cells (5–7, 13). The results of these studies are also shown in Table 1.

Overall, the findings of the preclinical studies corroborate the notion that PSIS-ECM exhibits minimal immunogenicity. Among the nine studies that entailed histological assessments of explants, eight reported no signs of inflammation or inflammatory reactions (5, 7–11, 13, 14). The singular exception was the study by Van Rijswijk et al. (12), where chronic inflammation and fibrosis were noted. Tissue remodelling, which involves the infiltration of various cells, such as fibroblasts, smooth muscle cells, adipocytes, and endothelial cells, along with the processes of neovascularization and reendothelialization, has been documented in six studies (8–10, 13, 14). However, Van Rijswijk et al. merely observed limited indications of tissue remodelling. In myocardial repair, the use of PSIS-ECM patches appears to enhance regeneration, thereby improving post-infarction cardiac function (5–7, 10, 11, 13, 15). This effect is particularly notable when PSIS-ECM is infused with either growth factors or cardiac cells (5–7, 13).

Since its initial clinical application, PSIS-ECM has found extensive use across a broad spectrum of cases, including pericardial reconstruction (16), valve repair or replacement (17–20), diverse congenital heart defects (17–19, 21–27), vascular repair (28–35), myocardial repair (36–39), and endocarditis (29, 40–43). The utilization of PSIS-ECM has extended to both adult and pediatric patients. Table 2 presents the outcomes of case studies conducted within the past decade.

The case study results indicate that pediatric patients experience less favorable outcomes and a higher rate of reintervention compared to adults. Of the nine pediatric cases reviewed, five (55%) presented significant complications, such as vessel stenosis, valve regurgitation, or atrial and ventricular dilatation, all of which required additional surgical or interventional cardiology procedures (18, 19, 22, 23, 25). In contrast, adult cases demonstrated fewer complications, with only four out of 19 cases (21%) reporting issues such as regurgitation, pseudoaneurysm formation, patch dehiscence, or early patch failure necessitating reoperation (27, 32, 37, 42).

Two specific case studies (25, 37) raised concerns about the durability of the PSIS-ECM patch. In one case, Mously et al. reported that a PSIS-ECM patch used for ventricular septal defect repair in a 58-year-old woman following myocardial infarction failed within seven months due to nearly complete reabsorption. Similarly, Rodrigues et al. observed almost total disintegration of the PSIS-ECM patch within just one month post-surgery in a 5-year-old patient with Tetralogy of Fallot and hypoplastic pulmonary arteries. These findings highlight potential weaknesses in using PSIS-ECM patches, particularly in pediatric patients, where rapid degeneration may lead to early failure.

Explants were available for histological analysis in 12 case studies (16–19, 23, 25, 26, 30, 32, 33, 42, 43). Among these, three case studies (16, 30, 33) documented a positive remodelling process with minimal or no signs of inflammation. Conversely, nine case studies (17–19, 23, 25, 26, 32, 42, 43) reported mild to severe chronic inflammation with no or minimal signs of remodelling. Additionally, Nezhad et al. observed calcification of the explant. Abu Saleh et al. compared a PSIS-ECM explant, which had been *in vivo* for 14 months, to an unused PSIS-ECM patch, revealing nearly identical cellularity and organization.

Table 3 presents the results of clinical cohort studies using PSIS-ECM in cardiovascular surgery over the last decade. The majority of these studies were retrospective in design (44–67), corresponding to evidence level III, with a primary focus on pediatric populations. Only three studies were conducted prospectively, two involving pediatric populations and one involving adult populations (68–70), placing them at evidence level II.

As presented in Table 3, PSIS-ECM has been employed for diverse purposes in clinical settings. These include various congenital heart defects (45, 46, 49–53, 55, 57–63, 66–69), vascular repair/reconstruction (54, 62, 64, 70), valve repair/replacement (47, 48, 56, 60, 61, 65), and complications arising from myocardial infarction (44).

Of the 27 clinical cohort studies, 13 (45, 46, 49, 51–53, 56, 58–60, 63, 68, 69) incorporated histological examination of explants, all of which demonstrated inflammation. The intensity and type of inflammation displayed varied among the studies, with the majority (45, 46, 49, 51–53, 56, 63) indicating chronic inflammation. This was characterized by inflammatory cell types, including macrophages, lymphocytes, eosinophilic cells, and giant cells. Furthermore, several studies have shown calcification (51–53, 60, 69) of the PSIS-ECM patch. No, or minimal signs of regeneration or remodelling were observed in the 13 studies.

A common outcome assessed across many of the studies is the reintervention rate. While specific studies report a relatively low reintervention rate of 4.5% (64), others report a significantly higher rate of 87.5% (61). Four studies (56, 61, 62, 66) are particularly noteworthy, given that they are comparative studies in which PSIS-ECM patches were compared to autologous pericardium patches.

In the study by Hu et al., 87.5% (7 out of 8) of their patients (mean age 13.63 ± 6.5 years) who underwent aortic valve repair with PSIS-ECM necessitated reoperation due to severe aortic regurgitation. In contrast, only 37.5% (11 out of 30) of the patients (mean age 10.25 ± 6.56 years) undergoing the same procedure with autologous pericardium required reoperation due to moderate/severe aortic regurgitation (61). The follow-up period for this study was five years.

Kelley et al. observed that 32% (8 out of 25) of their patients who underwent anterior leaflet augmentation of the mitral valve using PSIS-ECM required reoperation due to severe regurgitation. Conversely, none of the patients undergoing the same procedure with autologous pericardium required reoperation (56). The mean age in both groups was 62.6 ± 12.2 years, and the follow-up for this study extended up to 12 months.

Ebert et al. presented that 16.2% (6 out of 37) of their patients who underwent pulmonary artery reconstruction with PSIS-ECM needed reintervention. However, only 7.3% (3 out of 41) required reintervention among the patients undergoing the same procedure utilizing autologous pericardium. The median age in both groups was 12.1 months (5.6–59.5) (62).

Weis et al. noted that 55.5% (10 out of 18) of patients who underwent corrective repair of Tetralogy of Fallot or an Arterial Switch Procedure with PSIS-ECM required reoperation. Of these, 70% (7 out of 10) were attributed to patch failure. In comparison, 30.6% (11 out of 30) of the patients undergoing the same procedure with autologous pericardium required reoperation. Of these 11 patients, 27.3% (3 out of 11) were attributed to patch failure (66). The follow-up periods were 47 months for patients treated with PSIS-ECM and 121 months for patients treated with autologous pericardium. The median age of the study population was 11 months (2–60).

Among the case studies, less favorable outcomes and higher reintervention rates were evident in pediatric cases. Interestingly, this trend was not apparent in the clinical cohort studies. In 18 out of 27 clinical cohort studies, the reintervention rate was used as an outcome measure; five investigated adult patient populations, and thirteen studied pediatric patient populations. Three out of five (60%) (48, 54, 56) clinical cohort studies investigating adult patient populations reported reintervention rates exceeding 15%. Comparably, in nine out of thirteen (69.2%) (49, 50, 53, 60–62, 66, 68, 69) clinical cohort studies, pediatric patient populations presented reintervention rates greater than 15%.

## Discussion

Since the early 2000s, preclinical studies on the application of PSIS-ECM in experimental cardiovascular surgery have been conducted. Preliminary findings showed substantial promise, indicating that this biomaterial possessed characteristics indicative of an ideal material for cardiovascular tissue repair and cardiovascular tissue engineering. The material exhibited remodelling properties, absorbability, lack of immunogenicity, and potential to promote native tissue growth (3). These findings ultimately led to the first clinical application of PSIS-ECM in 2010. Since then, it has been applied in numerous clinical applications within cardiovascular surgery.

Manufacturers of CorMatrix claim that PSIS-ECM is a bioscaffold that facilitates native tissue growth and remodeling. This notion was upheld by early preclinical studies documenting remodelling, infiltration of host cells, endothelization, and neoangiogenesis (3). Recent preclinical studies conducted over the past decade have further corroborated this claim. Across all preclinical investigations that performed a histological assessment of explants featured in this review, the histological examination consistently indicated tissue remodeling with host cell infiltration of mesenchymal cells, adipocytes, fibroblasts, endothelial cells, and cardiomyocytes. Additionally, the studies disclosed neoangiogenesis, endothelialization, and tissue organization similar to native tissue (5, 7–11, 13, 14).

Contrary to expectations, these findings have scarcely been reproduced in clinical settings. Thirteen clinical cohort studies conducting histological analysis of explants were reviewed. Among these, twelve studies reported minimal or no infiltration of host cells and found no evidence of remodeling in the explants to resemble native tissue (18, 45, 46, 49, 51–53, 56, 59, 60, 63, 68). Cox et al. stand as the sole exception, presenting evidence of neo-intima, endothelialization, and the infiltration of smooth muscle cells into the graft (58).

The differences in tissue remodelling between animal models and humans are poorly understood, particularly in PSIS-ECM scaffold studies. One possible explanation is the scaffold's xenogeneic nature, which may provoke different responses in human patients. However, the remodeling process has been consistently observed in xenogeneic animal models, such as sheep, rats, and mice (5, 7–11), suggesting that additional factors may contribute to the discrepancy.

For example, preclinical studies typically use healthy animals with normal physiology, while patients in clinical studies often have significant underlying pathologies. Since variations between healthy and pathological conditions are likely to influence tissue regeneration, it is essential to investigate whether the positive outcomes seen in preclinical models can be replicated in pathological animal models. Such studies could provide more translational and accurate insights into how PSIS-ECM scaffolds perform in clinical scenarios.

Despite preclinical studies demonstrating that PSIS-ECM exhibits properties associated with tissue remodelling and the potential for native tissue growth, the prospects of complete reabsorption and replacement with native tissue in humans are questionable. The skepticism arises from the fact that 12 out of 13 clinical studies in the current review report either no or minimal infiltration of host cells and no tissue remodelling when PSIS-ECM is employed in humans.

The outcomes presented in this review imply a distinct variation in the immunogenic response to PSIS-ECM between animal subjects and humans. Out of nine preclinical studies incorporating histological assessment of explants, eight reported no signs of inflammation or inflammatory reaction (5, 7–11, 13, 14). Contrarily, all 13 clinical studies that conducted histological analysis of explants demonstrated inflammation of varying intensity, with the majority displaying chronic inflammation (45, 46, 49, 51–53, 56, 58–60, 63, 68, 69). Similar findings have been reported by Nezhad et al. (3).

PSIS-ECM has undergone decellularization, lowering the risk of triggering the human immune system. Nonetheless, PSIS-ECM is xenogeneic when employed in humans, thus potentially expressing epitopes that can activate immunological pathways and induce inflammatory reactions. Interestingly, several preclinical studies using PSIS-ECM in various xenogeneic animal models, such as sheep (8–12, 15, 71–74), rats (7, 75), mice (5, 6), and calves (76, 77), yielded results without signs of inflammation. Only three preclinical studies utilizing PSIS-ECM in a xenogeneic model since the 2000s have identified inflammation upon histological examination of explants (12, 78, 79).

The difference in the immunogenic response to PSIS-ECM between animal subjects and humans requires further investigation. Rosario-Quinones et al. suggest that the variance in immunogenicity may stem from PSIS-ECM expressing gal-a epitopes, to which humans possess natural antibodies (49). However, this is only speculation; further inquiry is warranted to amplify our knowledge.

We found a considerable variance in the reported reintervention rates among the clinical cohort studies included in the present review. Reintervention rates may be influenced by a variety of factors, including the expertise and proficiency of the surgeon conducting the procedure, the characteristics of the patient population, the anatomical site where the PSIS-ECM patch is applied, the immunological response to the PSIS-ECM patch, the number of layers of the PSIS-ECM patch, and the duration of the follow-up period.

The anatomical site of PSIS-ECM patch implantation appears to be particularly important. Padalino et al. concluded that PSIS-ECM is characterized by good late outcomes for septal and aortic wall reconstruction, but suboptimal functional late performance when used to reconstruct semilunar valve leaflets or small pulmonary arteries (68). Weis et al. reached a similar conclusion, declaring that PSIS-ECM performed well for the closure of atrial and ventricular defects. In contrast, using PSIS-ECM for arterial vessel reconstruction and valve reconstruction was associated with higher reintervention rates compared to standard autologous pericardium (66). Witt et al. correspondingly noted that PSIS-ECM performed well in pediatric cardiovascular reconstructions, particularly those with septal defects or vascular patch augmentation remote from the outflow tract (45). Consistent with these observations, three out of five clinical cohort studies (55, 57, 64) reporting reintervention rates below 15% utilized PSIS-ECM for septal defects or extracardiac vessel repair. Witt et al. and Haney et al., who also report a reintervention rate of less than 15%, applied PSIS-ECM at various sites, including valves, the interventricular septum, and extracardiac vessels. However, the majority of the sites were septal or extracardiac vessels. The clinical studies in this review reporting reintervention rates greater than 15% primarily employed PSIS-ECM for valve reconstruction, valve replacement, or pulmonary artery repair (48–50, 53, 56, 60–62, 66, 68, 69). The available evidence suggests that PSIS-ECM performs more reliably in low-pressure, extracardiac or septal settings. In contrast, applications involving valve or outflow tract reconstructions are associated with higher rates of reintervention. However, due to limited data granularity, inconsistent reporting, and the absence of prospective stratification, further generalization is not currently supported.

The disparity in reintervention rates across various anatomical locations may be related to variations in hemodynamic pressure exerted on the patches. Hemodynamic pressures and the mechanical stresses they impose on tissues vary among anatomical sites, and it is established that mechanical stress influences the regeneration processes (80). Thus, the hemodynamic conditions near the interventricular septum could promote regeneration more efficiently than those near the valve locations and the pulmonary artery site. Despite this, the precise relationship between the positioning of the PSIS-ECM patch and the reintervention rate is yet to be fully understood.

As previously addressed, all included clinical cohort studies assessing the histology of explants document varying degrees of inflammation. It remains to be determined whether the inflammatory response resulting from the implantation of PSIS-ECM patches contributes to the deterioration and eventual malfunction, thereby necessitating reintervention. A paradox arises in this context. Inflammation is an inherent aspect of the recellularization process, and it remains unclear whether the observed inflammation would have contributed to regeneration had the follow-up period been extended. However, this cannot be determined, as patch failure prevents the possibility of prolonging the follow-up period.

Another potential factor contributing to the variability in clinical and histological outcomes is the heterogeneity in the source and preparation method of the PSIS-ECM scaffolds. While some studies explicitly stated that they used commercially available products, such as CorMatrix®, others did not specify whether the scaffold was commercially sourced or produced in-house. Moreover, very few studies provided technical details about the decellularization protocols or batch-specific characteristics. This lack of standardization complicates the interpretation of outcomes across studies and may partially account for the discrepancies in inflammation, remodeling, and rates of reintervention. Future studies should report the source and preparation details of the scaffold more rigorously to enable better reproducibility and cross-study comparison.

The number of layers in PSIS-ECM might affect the surgical outcomes. It is speculated that the risk of thrombosis between the layers, leading to patch failure and the need for reintervention, increases with the number of layers in the patch. Furthermore, the mechanical stresses exerted on the patches are contingent upon the number of layers in the patch. As mechanical stress is known to influence the regeneration process, the number of layers could potentially affect the regeneration process and, therefore, the reintervention rate.

Haney et al. are the sole researchers to have conducted a clinical comparative analysis of the outcomes of utilizing 2-ply vs. 4-ply PSIS-ECM in cardiovascular surgery (67). Surgical reintervention was required in 2.1% of patients treated with 4-ply PSIS-ECM, while 6.3% of patients treated with 2-ply PSIS-ECM underwent surgical reintervention. Percutaneous intervention was performed on 6.4% of patients treated with 4-ply PSIS-ECM. In comparison, 9.4% of patients treated with 2-ply PSIS-ECM necessitated percutaneous intervention. However, the sample size was considerably larger for 4-ply PSIS-ECM (*n* = 372) compared to 2-ply PSIS-ECM (*n* = 32). Additionally, conclusions should not be drawn from a single-center study. Accordingly, further research is required to unfold the impact of various layers in the PSIS-ECM patches.

As noted in the results section, case studies demonstrated less favorable outcomes in pediatric patients than in adults. However, this trend was not apparent in the clinical cohort studies. Given that clinical cohort studies provide stronger evidence than case studies, we do not consider the correlation between age and outcomes significant.

The source of the high reintervention rate is likely multifactorial, making it challenging to determine the precise causative factors. Nonetheless, it is worrisome that most clinical studies in this review reported a reintervention rate greater than 15%. Based on their findings from clinical cohort studies reporting reintervention rates exceeding 10%, Nezhad et al. raised ethical concerns about conducting more extensive clinical studies using PSIS-ECM in the pediatric population (3). Thus, our findings further emphasize these concerns articulated by Nezhad et al.

As indicated in the results section, only three studies included in this review are classified as evidence level II, with the remainder falling under evidence levels III or IV. While this may not pose a significant issue due to the number of included studies with similar findings, the overall strength of the evidence would have been greater if the majority of studies had been at evidence level II.

A limited number of comparative studies have been conducted to date, comparing PSIS-ECM to well-established materials, such as autologous pericardium, xenogeneic pericardium, and synthetic materials (56, 61, 62, 66, 81). However, these studies were undertaken retrospectively. Thus, additional, preferably prospective and comparative, research is required. Furthermore, the significance of the layer count of the PSIS-ECM material required further evaluation. Investigating the vacuum-pressed iteration of PSIS-ECM by CorMatrix would also be of interest. Considering the high reintervention rate in many clinical cohort studies reviewed in this present review, conducting these studies in a xenogeneic animal model should precede the transition to a clinical setting, preferably a pathological animal model.

Preclinical studies (5–7, 10, 11, 13, 15) and clinical case studies (36, 38) have shown that applying a PSIS-ECM patch to cover a damaged area post-acute myocardial infarction can improve heart regeneration. Therefore, further preclinical research and clinical cohort studies on this topic are warranted. Additionally, preclinical trials indicate that this effect is enhanced when the PSIS-ECM is augmented with growth factors or cardiac cells (5–7, 13). However, limited knowledge exists, necessitating further research on the impact of augmenting PSIS-ECM with diverse growth factors and cells.

## Limitations

Our literature search was confined to two databases, which could have excluded relevant studies. Additionally, there is great variability in the indications and outcome measures across the studies included in this review, which may lead to less generalizable conclusions. Furthermore, many studies did not specify whether the PSIS-ECM used was commercially available or laboratory-prepared, nor did they provide details on the decellularization protocol or characterization of the scaffold. This variability in scaffold origin and preparation introduced additional heterogeneity, which could have influenced the observed histological and clinical outcomes.

## Conclusion

Preclinical studies over the past decade have consistently demonstrated that PSIS-ECM possesses several attributes of an ideal biological scaffold. However, these theoretical advantages have yet to be replicated in clinical applications. Clinical studies have reported significant limitations, including inflammation, calcification, a lack of remodelling, and minimal host cell infiltration. Furthermore, reintervention rates exceeding 15% in many clinical cohorts highlight concerns regarding the long-term durability of PSIS-ECM in cardiovascular surgery.

Despite these challenges, the selective application of PSIS-ECM, such as in septal defects or extracardiac vascular pathologies, has shown comparatively better outcomes with low reintervention rates. These findings suggest that its clinical use should be restricted to specific anatomical sites where the risk-benefit profile is more favourable.

The disparity between preclinical and clinical outcomes underscores the need for further investigation. Future research should prioritize understanding the mechanisms underlying these discrepancies, with a main focus on inflammation, calcification, and remodelling. To address these questions, we propose using pathological xenogeneic animal models as a bridge to improve translational insights before advancing to additional clinical studies. Such an approach may enhance the predictive value of preclinical findings and inform the safe and practical application of PSIS-ECM in cardiovascular repair.

## Data Availability

The original contributions presented in the study are included in the article/Supplementary Material, further inquiries can be directed to the corresponding author.
